# Challenges and Controversies Concerning Neisseria gonorrhoeae-Neutrophil Interactions in Pathogenesis

**DOI:** 10.1128/mBio.00721-21

**Published:** 2021-06-01

**Authors:** Alison K. Criss, Caroline A. Genco, Scott D. Gray-Owen, Ann E. Jerse, H Steven Seifert

**Affiliations:** a University of Virginia School of Medicine, University of Virginia, Global Infectious Diseases Institute, Charlottesville, Virginia, USA; b Tufts University School of Medicine, Boston, Massachusetts, USA; c Temerty Faculty of Medicine, University of Toronto, Toronto, Ontario, Canada; d Uniformed Services University, Bethesda, Maryland, USA; e Northwestern University Feinberg School of Medicine, Chicago, Illinois, USA; University of Illinois at Chicago

**Keywords:** *Neisseria gonorrhoeae*, gonorrhea, neutrophils

## Abstract

The bacterium Neisseria gonorrhoeae (Ngo) is the main cause of the sexually transmitted infection gonorrhea. The global incidence of 87 million new Ngo infections each year, rising infection rates, and the emergence of Ngo strains that are resistant to all clinically recommended antibiotics have raised the specter of untreatable infections (M. Unemo, H. S. Seifert, E. W. Hook, III, S. Hawkes, et al., Nat Rev Dis Primers 5:79, 2019, https://doi.org/10.1038/s41572-019-0128-6). Given their abundance in symptomatic disease, neutrophils are central to both Ngo infection and consequent damage to host tissues. This article highlights present knowledge and the main open questions about Ngo-neutrophil interactions in immunity versus disease pathogenesis.

## OPINION/HYPOTHESIS

## GONORRHEA

Neisseria gonorrhoeae (Ngo) naturally infects the mucosae of the human urethra, cervix, pharynx, and rectum (1). A purulent exudate consisting almost entirely of neutrophils and Ngo defines symptomatic infections. While up to 40% of cervical infections and most pharyngeal and rectal infections are clinically inapparent, neutrophil influx can still occur (1). Serious sequelae occur when there is damage to colonized tissues, with neutrophils implicated in this response. Ascending urethral infection in men can result in inflammation of the testicle (epididymitis) (2). In women, 18 to 20% of untreated cervical infections transit to the endometrium and may cause pelvic inflammatory disease (PID). Gonorrhea is associated with adverse pregnancy outcomes, and babies born to infected mothers have a 30 to 45% increased risk for acute conjunctivitis. Disseminated gonococcal infection (DGI) can occur in either sex and can manifest as dermatitis, infectious arthritis, or endocarditis. While neutrophils’ participation in Ngo pathogenesis is well recognized, many questions remain about where, when, and how they contribute to infection.

## PROPERTIES OF N. GONORRHOEAE

Several notable features of Ngo complicate the study of Ngo-neutrophil interactions. First, the cell wall and outer membrane of Ngo have potent inflammatory potential. The hexa-acylated lipid A portion of the Ngo lipooligosaccharide (LOS) is a strong agonist for Toll-like receptor 4 (TLR4) (3), its surface-exposed lipoproteins are recognized by TLR2 (4), it sheds abundant peptidoglycan fragments during normal growth that are recognized by NOD-like receptors (5), it releases heptose phosphate sugar intermediates of LOS biosynthesis that stimulate the TNF receptor associated factor (TRAF)-interacting protein with forkhead-associated domain, and some strains have a type IV secretion system that releases methylated DNA (6). While Ngo activates these pattern recognition receptors in macrophages and transformed cell lines, how neutrophils respond to these TLR agonists and contribute to the response to Ngo need evaluation.

Second, Ngo has an extensive capacity to phase and antigenically vary three surface components that modulate interaction with neutrophils, the type IV pilus (T4p), opacity proteins (Opa), and LOS (1). While phase variation turns ON or OFF expression of the variable component, antigenic variation modifies the sequence and, in some cases, the function of surface molecules. Thus, every population of Ngo is a mixture of cells that express none or different variants of these components, each of which can alter Ngo phenotypes and/or host cellular response. Some of the conflicting reports of Ngo interaction with neutrophils may result from undefined differences in the Ngo population used, even if in the same strain background. Additionally, since phase and antigenic variation are uncontrolled, selection for different phenotypes can occur *in vivo* by factors that nonhuman models of infection do not fully replicate. The use of laboratory-engineered bacteria that limit phase or antigenic variation and experimental systems that incorporate possible selective host factors may help resolve these conflicts.

Third, Ngo is a fastidious bacterium that requires rich medium for *in vitro* growth. However, these conditions may downregulate factors that support human mucosal infection and Ngo-neutrophil interactions. For instance, iron, which is limiting *in vivo* but abundant in most growth media, suppresses the expression of many Ngo virulence factors (7). Both iron and oxygen, the latter of which varies in different body sites, can influence Ngo gene expression and the efficacy of neutrophil oxidative killing.

Fourth, differences in male and female hosts and anatomical sites may alter Ngo-neutrophil interactions. For example, Ngo shows higher expression of the Mtr efflux pump that defends against antimicrobial peptides in the urethra compared to the cervix (8), and efflux pump mutants are more common with cervical isolates (9). Beyond features intrinsic to Ngo, the local microbiota may also place selective pressures on Ngo that influence its interactions with neutrophils. For instance, Ngo adds sialic acid to its LOS as a defense against complement-mediated killing and antimicrobial peptides, but sialidases expressed by the cervicovaginal microbiota can remove these sugars (10).

## MODELS TO STUDY GONOCOCCAL PATHOGENESIS

Ngo-neutrophil interactions have been mainly studied using isolated neutrophils, which allows the conditions of infection to be directly manipulated. Many researchers have used neutrophils that are isolated from human peripheral blood, which maintain the full migratory, phagocytic, and antimicrobial capacity of neutrophils *in vivo.* However, primary human neutrophils have short lives and are not genetically modifiable, and unknown factors encountered during isolation or intrinsic to the donor can alter their response to Ngo, making experimentation with these cells technically challenging. Neutrophil-like cell lines, including differentiated HL-60 cells, are surrogates that are tractable to genetic manipulation and retain many signaling pathways of neutrophils; however, they cannot make certain antimicrobial granule subsets or kill Ngo. Murine primary neutrophils can be genetically manipulated; however, mouse neutrophils are less efficient at certain antimicrobial responses than human neutrophils (11, 12). The most critical difference is that murine neutrophils do not express human CEACAMs, which mediate the nonopsonic phagocytosis of certain Opa^+^ Ngo and concomitant CEACAM3-dependent neutrophil activation. Isolation of neutrophils from transgenic mice expressing human CEACAM3 has helped address this issue (see below).

The physiological state of neutrophils also affects their interactions with Ngo. For instance, isolated neutrophils in suspension are poor at phagocytosing Opa-negative Ngo in the absence of serum opsonins, but adherent neutrophils can (13). We need to define Ngo-neutrophil interactions in systems that mimic *in vivo* infection, such as neutrophils that have transmigrated across polarized human endocervical cells. Infection models outside humans or other animals may not account for factors that are present *in vivo*, such as CMP–*N*-acetylneuraminic acid (NANA) for LOS sialylation, which Ngo acquires from the host to protect itself from opsonic and Opa-mediated phagocytic uptake (14).

There are two well-established mouse models used to examine Ngo colonization and neutrophil interactions: vaginal infection of estradiol-treated wild-type mice, which allows lower genital tract colonization, or of mice that express human transgenes known to be important for Ngo pathogenesis (15). Both models feature neutrophil recruitment at sites of Ngo colonization and have been critical to reveal Ngo-neutrophil interactions in tissues. Recently, a transcervical infection model has been developed to examine how Ngo and neutrophils interact in the upper genital tract when under the influence of estradiol or progesterone (15). These models do not fully recapitulate human infection—for instance, in mice the type IV pilus is dispensable for infection, whereas pilus expression is strongly selected for in humans. However, these models, combined with the ability to deplete neutrophils and murine genetic tools, will allow new hypotheses about Ngo-neutrophil interactions to be generated.

The human challenge model involves urethral infection of male volunteers with *in vitro*-grown Ngo and examination of Ngo shed in urine or collected by urethral swabs (16). The human challenge model is limited by the small number of subjects in each trial, a short (5-day) period of colonization, the need for immediate treatment if a purulent exudate forms, and the inability to collect Ngo or human cells directly from mucosal surfaces. However, this model has importantly defined factors that are required for Ngo to survive in the male urethra.

## NEUTROPHIL CONTRIBUTIONS TO Ngo PATHOGENESIS

Upon experimental infection of the human male urethra, inflammatory cytokines, including the neutrophil chemotactic factor IL-8, are detected in blood and urine, leading to the detection of neutrophils in urine or urethral swabs on average 3 days after inoculation (7). Ngo pathogen-associated molecular patterns like peptidoglycan and LOS are potent triggers of pattern recognition receptor-driven proinflammatory responses that result in neutrophil recruitment and disruption of epithelial barrier integrity (17). Moreover, gonococcal Opa protein binding to CEACAM3 stimulates a feed-forward inflammatory program in CEACAM3-transgenic mice, with the release of additional cytokines such as MIP-1 and MIP-2 to drive further neutrophil recruitment (18). Two eicosanoid lipids, hepoxilin A3 made by epithelial cells and leukotriene B4 made by neutrophils, coordinate the movement of primary human neutrophils across human endocervical epithelial cell monolayers following Ngo infection (19). The relative importance of these factors for neutrophil recruitment and the ability to phagocytose and kill Ngo has not been fully explored.

Recruitment to the infected epithelium not only allows neutrophils to gain access to luminal bacteria but also prevents Ngo accumulation within the tissues. Neutrophils fill the uterine lumen when diestrus-phase mice are transcervically infected with modest numbers of Ngo, but tissue ulceration occurs with higher bacterial burdens (18), consistent with the recruitment and activation of neutrophils causing much of the secondary sequelae associated with upper reproductive tract infection. The stage of the female reproductive cycle is one host property that significantly affects Ngo pathogenesis, since PID often arises in women shortly after the onset of menses (20). Notable in this regard, estrus-stage mice also display a proinflammatory cytokine response following inoculation with Ngo but have little neutrophil infiltration into the infected uterus (18). Also, neutrophil depletion leads to higher loads of Ngo in mice transcervically infected during diestrus but does not impact the bacterial burden when the depleted animals are infected at estrus; whether this stems from the difference in neutrophil recruitment or a direct hormonal effect on neutrophil function and/or relates to hormone-induced changes in mucus and other factors remains unclear. It also needs to be determined if there is an impact of hormonal cycling at other infected mucosal sites and whether there is a role of hormones in male urethral infection.

## Ngo Opa VARIANT EXPRESSION AND NEUTROPHILS

Many of the ∼11 Opa variants encoded by each Ngo isolate bind to human CEACAM1, CEACAM3, and/or CEACAM6 on neutrophils (21). CEACAM3 has a cytoplasmic immunotyrosine-based activation motif (ITAM) that is required for reactive oxygen species (ROS) production and release of primary granules, and the majority of Opa^+^ bacteria that bind CEACAM3 are killed inside neutrophils (13). Primary Ngo isolates from humans predominantly express Opa proteins that bind CEACAM1 but not CEACAM3 (22), implying a selective pressure against bacteria expressing CEACAM3-binding Opa variants. An ongoing goal of the field is to develop strains that express different combinations of these Opa variants, both alone and with other adhesins that promote neutrophil binding, coupled with the implementation of enhanced tools for investigating signaling in otherwise terminally differentiated neutrophils, to better understand how receptor-ligand interactions affect Ngo phagocytosis and neutrophil antimicrobial and proinflammatory activities.

Gonococci that have phase-varied off Opa protein expression (Opa-negative bacteria) are phagocytosed by human neutrophils in the absence of serum opsonins, though less efficiently than is Opa^+^ Ngo. This response requires the human neutrophils to be adherent and treated with interleukin-8; neutrophils in suspension do not bind or phagocytose unopsonized Opa-negative bacteria. Unlike CEACAM3-binding Opa^+^ Ngo, Opa-negative bacteria do not induce ROS but instead inhibit ROS production from a variety of stimuli including CEACAM3, do not stimulate efficient primary granule release, and survive significantly better inside human neutrophils (23, 24). Notably, Opa-negative Ngo opsonized with IgG behaves similarly to CEACAM3-binding Opa^+^ bacteria, effects attributed to signaling through Fc receptor and CEACAM3 ITAM domains. In contrast, human serum (complement)-opsonized bacteria largely phenocopy Opa-negative Ngo. These marked differences in the outcome of infection highlight why the experimental conditions must be carefully defined and explain why differences in infection methodology can so potently affect measured outcomes. Further, it is important to consider if the experimental systems mimic early stages of infection where nonopsonic uptake by neutrophils is likely more important than it is during periods of inflammation, when complement and cross-reactive antibodies from serum exudates become present on mucosal surfaces, and to consider how complement deposition influences Ngo-neutrophil interactions early in infection.

Given current efforts to develop a gonorrhea vaccine, how Ngo-binding antibodies contribute to bacterium-neutrophil interactions warrants renewed evaluation. Mice immunized with meningococcal serogroup B vaccine and challenged vaginally with Ngo exhibit vaginal neutrophil recruitment and make antibodies that bind Ngo and promote bacterial clearance, implying opsonophagocytosis could contribute to protection (25). The 2C7 anti-LOS vaccine prototype promotes opsonophagocytic killing of the “3-Hex/G+” Ngo glycotype by primary human neutrophils (26); however, *in vivo* depletion of neutrophils did not affect the efficacy of 2C7 against vaginal Ngo colonization (27). Since correlates of protection for vaccines against gonorrhea remain unresolved, the contribution of opsonophagocytic killing by different vaccine candidates should remain a priority, along with development of standardized assays for this aspect of pathogenesis.

## EVASION OF NEUTROPHIL BACTERICIDAL RESPONSES

Neutrophils produce antimicrobial components including reactive oxygen species (ROS), antimicrobial peptides and proteins, proteases, and nutritional immunity proteins. Understanding how gonococci survive following neutrophil interaction is challenging because the recovery of bacteria requires neutrophil lysis, which can release toxic granular contents that can inadvertently impact gonococcal viability. Detailed studies from multiple groups have shown that Ngo is resistant to neutrophil-derived ROS, even under conditions where ROS is abundant (e.g., CEACAM3-binding or IgG-opsonized Ngo) and antioxidant defenses are absent (e.g., Ngo mutants defective in catalase and superoxide dismutase) (28). *In vitro* assays with purified components have identified gene products that enable Ngo defense against the nonoxidative components of neutrophils, but in many cases, their contribution to survival from neutrophils remains untested (13). Neutrophil extracellular traps (NETs) contain a subset of antimicrobial products and are generated in neutrophils exposed to Ngo *in vitro*, but whether, where, and how NETs are made *in vivo* in response to Ngo, and the relevance of these structures to control of Ngo infection, are currently unknown. Imaging animal or tissue models where neutrophils and Ngo interact in real time would help to understand how efficiently neutrophils engulf and/or entrap Ngo in NETs, and how each contributes to the outcome of infection.

When Ngo is exposed to neutrophils *in vitro*, a portion of the inoculum is killed over time. The inherent variability of Ngo can affect the extent of killing, as can differences in polymorphonuclear leukocyte (PMN) responsiveness. A systematic exploration of how each of these factors contributes to the interactions and how Ngo responds to these factors is needed. The killing of Ngo by neutrophils is increased by the inactivation of components including T4p, efflux pumps, nutrient access, LOS sialylation, and lysozyme resistance factors, but their relative importance and their potential to act synergistically need investigation (13).

A key unresolved question in the field is how the susceptibility of Ngo to neutrophils changes during infection. There is indirect evidence for Ngo replication inside neutrophils, but when, where, and how this occurs need investigation. Ngo appears to modulate PMN apoptosis, but there are reports of both gonococcus-induced inhibition and enhancement of PMN apoptosis (1). Finally, in gonorrheal exudates, only a subset of neutrophils have intracellular Ngo. Whether this subset is functionally different from others in the population, or if this reflects different stages of neutrophil-Ngo interactions, remains to be determined.

## IMMUNITY VERSUS IMMUNOPATHOGENESIS: INFLUENCE OF NEUTROPHILS

Perhaps the largest unresolved question in the field is whether the neutrophil-driven inflammatory response most benefits the host or the pathogen. The central paradox of gonorrhea is that even with an intense, sustained inflammatory response to Ngo, the bacteria persist and there is no protective adaptive immune response. This is a key point because repeated infection of a core group of individuals who engage in high-risk sexual behavior allows persistence of sexually transmitted infections within a population. Phosphoethanolamine modification of gonococcal LOS, which does not occur in most commensal *Neisseria*, increases proinflammatory cytokine production in both human tissue culture cells and experimentally infected mice, implying that inflammation may be beneficial for the pathogen (1). Moreover, Ngo infection of the female mouse genital tract elicits a Th17-biased response (29), which promotes neutrophil recruitment to the affected tissues but suppresses the development of memory responses (15). Men and women with uncomplicated gonorrhea also show Th17 responses, but the details of innate signaling during human infection need further study.

Tissue-specific differences among mucosal environments are likely to impact neutrophil responses to Ngo. In the vagina and cervix, neutrophils must respond to pathogens such as Ngo while also allowing the survival of the commensal flora and, during pregnancy, enabling implantation of the embryo and development of the fetus (30). The murine lower genital tract Ngo infection model uses estradiol, which also prevents the natural influx of neutrophils that occurs in the luteal stage of the reproductive cycle, implying that neutrophils help control infection (31). As noted above, transcervical Ngo infection of mice that mimics upper reproductive tract infection leads to responses ranging from an intense inflammation reminiscent of PID that occurs during the progesterone-driven diestrus state to an almost complete absence of neutrophil infiltration when the mice are in estrus (18). These findings align with the variability of neutrophil responses to Ngo in humans, and more generally with sex hormones modulating immune responses at mucosal sites (32). How hormones may also affect neutrophil functionality warrants further investigation.

## CONCLUSIONS

Ngo interactions with neutrophils are at the center of mucosal colonization, immunity, and disease. Yet given that N. gonorrhoeae is highly adapted to life in humans, undergoes extensive genotypic and phenotypic variation, and infects multiple different mucosal tissues, progress in understanding the role of Ngo-neutrophil interactions during infection is challenging. For instance, it remains unclear whether there is control of the neutrophilic response to Ngo by the bacteria to avoid the development of an adaptive immune response, or if this is a futile attempt by the immune system to combat infection. Adding to the complexity, recent studies show that neutrophils are not a homogeneous lineage, and subsets exhibit differences in antimicrobial activity, maturity, and cell death pathways, which could influence responses to Ngo (33). How Ngo-neutrophil interactions are influenced by pathogen factors, different responses of the various tissues that Ngo colonizes, the local microbiota, and the cytokine and hormonal milieu to which the bacteria are exposed and how these responses might damage host tissues are undoubtedly central to determining the outcome(s) of infection. Understanding these effects will have important implications for the development of host-targeted therapeutics to combat infection by drug-resistant strains and the selection of adjuvants for future gonococcal vaccines.
